# From Tragedy To Opportunity: Hospital-based Violence Intervention Programs May Address Root-Cause Health Disparities for Violent Traumatic Injury Patients

**DOI:** 10.1177/21501319251356386

**Published:** 2025-08-11

**Authors:** Khadija Brouillette, Joseph Gebru, Armaan K Malhotra, Tyler McKechnie, Husain Shakil, Joseph Tropiano, Adom Bondzi-Simpson

**Affiliations:** 1 McGill University, Montreal, QC, Canada; 2 Brock University, St. Catharines, ON, Canada; 3 University of Toronto, ON, Canada; 4 McMaster University, Hamilton, ON, Canada; 5 University of British Columbia, Vancouver, Canada

**Keywords:** trauma, violence, equity, community-based intervention, prevention

## Abstract

Violence is a growing public health issue that disproportionately affects low-income and racialized communities across North America. While trauma centers appropriately respond to acute violent injuries, many patients are discharged back into the same environments that put them at risk. Hospital-based Violence Intervention Programs (HVIPs) offer opportunities for trauma care to address the upstream root causes of violence. These programs engage patients during critical “teachable moments,” connecting them to staff with lived experience, along with social workers and community partners, providing personalized support such as mental health care, education, employment, and housing services. In Canada, several initiatives in Toronto and Winnipeg are examples of HVIPs assisting with reduction of repeat injury by intervening on root causes. The early results show reductions in re-injury, improved engagement in school, lower justice system involvement, and potential healthcare cost savings. This commentary explores Canadian HVIPs as a model for addressing health disparities linked to violence and considers how similar approaches can be adapted in other healthcare settings to better serve communities.

## Introduction

Violence has devastating public health costs and contributes to preventable early death, injury, and long-term morbidity.^1,2^ Across North America, gun violence is the leading cause of homicide, with firearm-related incidence steadily climbing since 2016.^3^ Globally, firearm-related mortality is highest among young adult men and strongly associated with race, ethnicity, and low educational attainment and opportunity.^4,5^ Given the frequency with which victims of violence interact with the healthcare system, hospitals, particularly trauma centers, present a key opportunity to intervene on the root causes of violence.

Trauma from violence demands a coordinated, systematic, and robust response that spans across several phases of care.^6,7^ Prehospital care focuses on stabilizing and transporting patients through emergency medical services to a trauma center. Once the patient is received, advanced resources, experienced personnel, and subspeciality care will focus on resuscitation, management, and definitive care of life-threatening injuries.^8^ However, while trauma centers are highly effective in acute medical intervention, the transition from hospital to community remains a critical gap in violence prevention and recidivism.^9^ Focus on discharge, rehabilitation, and re-integration may be critical to address root causes of violence.^10,11^

Hospital-based Violence Intervention Programs (HVIPs) are targeted programs that focus on reducing repeat violent injury in youth by addressing upstream social determinants of health (SDoH). HVIPs extend trauma care beyond physical healing to addressing broader structural factors that perpetuate cycles of violence, allowing for recovery and re-entry into society and the workforce.^7,12,13^

This commentary will examine HVIPs in Canada as a model for addressing violence-related health disparities to better support communities.

## Structural Drivers of Violence

**Figure 1.**
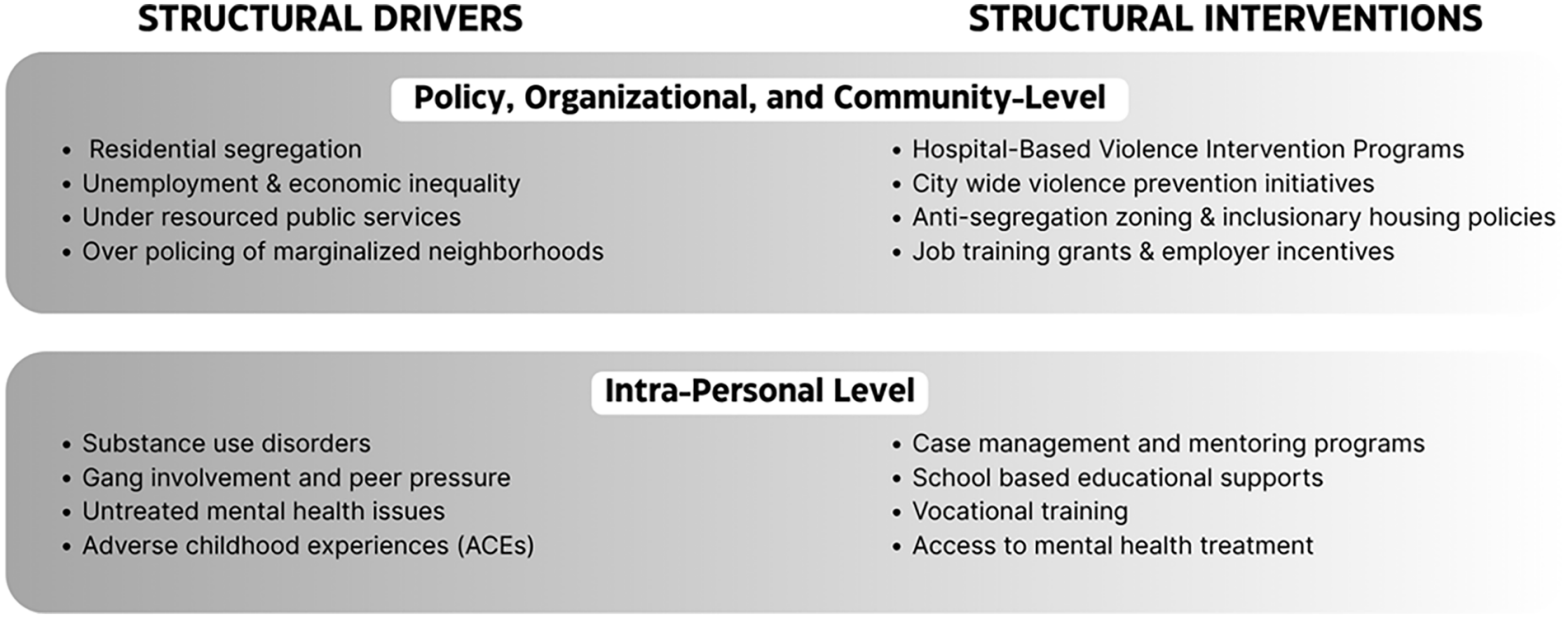
Examples of structural drivers and interventions in violence prevention.

Structural determinants, *not* individuals or culture, are the primary drivers of violence in society.^14^ These determinants shape the risk factors for violence. Literature suggests that structural determinants (such as economic instability, inadequate housing, and limited educational opportunities) create the conditions (such as substance use, domestic or community violence, and gang involvement) that lead to violent injuries.^14-17^ For example, communities facing poverty, unemployment, and racial segregation experience higher rates of violence and firearm injuries.^18-20^ However, interventions that target these root causes reduce violent crimes. In Philadelphia, a citywide trial of renovations to abandoned buildings found that installing working doors and windows led to a 13.1% reduction in gun assaults.^21^ Similarly, in Chicago, a youth summer vocational program resulted in a 45% decrease in violent-crime arrests^22^ and a school-based violence prevention program achieved a 15% median reduction in violent behavior.^23^ These findings highlight the profound impact of addressing the structural determinants of health which can lead to meaningful reductions in violence and enhance community safety at-scale (Figure 1).

## Hospital-Based Violence Intervention Programs in North America

HVIPs are public health initiatives launched in trauma centers that provide patients presenting with violent traumatic injuries, such as gunshot wounds, stabbings, or assaults, with immediate and long-term support.^12^ Built on the principle that violence is a public health crisis, HVIPs emerged in response to extremely high rates of recidivism for violent injuries, with rates ranging from 9% to 58%.^2,10^ Given that hospitals often become the first point of contact for victims of violence, the hospital setting presents an opportunity to “break the cycle”.^11^

In Winnipeg, Ontario, in 2016, Dr. Carolyn Snider pioneered the Community Emergency Department Violence Intervention Program (CEDVIP) initiative that laid the foundation for HVIPs in Canada.^24,25^ The underlying framework of CEDVIP was to focus on reducing repeat violent injury in youth by addressing upstream SDoH, such as education, employment, housing, and mental health support.^25,26^ In CEDVIP, youth presenting to the emergency department with violent injuries were met by a support worker with lived experience, who initiated “wrap-around care” for up to a year, including connection to community-based resources.^25^ The initial randomized control trial assessing the feasibility and efficacy of CEDVIP demonstrated a 10.4% absolute reduction in repeat violent injuries, along with improvements in education engagement and decreased justice system interactions.^26^ The success of CEDVIP revealed how HVIPs can break cycles of violence by addressing root causes, making it a model for expanding violence prevention programs in healthcare. Since then, trauma centers across Canada and beyond have implemented HVIPs to reduce trauma injury recidivism and support patients.^27^ Depending on local resources, HVIPs may offer mental health and substance use treatment, housing assistance, education, job training, employment, court advocacy, and victims of crime assistance.^12,28^ Below we describe two HVIPs developed in Canada’s most populous province – Ontario.

## Hospital-Based Violence Intervention Programs in Ontario, Canada

BRAVE program (Beyond Reasonable Approaches to Violence Eradication) and the THRIVE program (Toronto Hospital-based Reducing Injury from Violence Intervention and Evaluation) are two HVIPs located in the city of Toronto with the similar goals: to prevent reinjury and recidivism among young victims of violence (ages 14-29 years) through provision of trauma-informed care, mental health support, mentorship, and connections to community resources

Launched in 2020 at Sunnybrook Hospital, BRAVE responded to growing awareness of structural systemic issues such as limited access to education and employment and the impacts of systemic racism in Toronto. Funded by the City of Toronto, the program was launched to address the rising number of violent injuries among youth by taking an upstream approach.^29-31^ Since 2020, the program has achieved a 37% reduction in violence risk factors, with high patient engagement and reported improvements in motivation, focus, and recovery and expanded to two other hospital sites in the city.^32^ In 2023, THRIVE was launched at St. Michael’s Hospital.^31^ The development of these programs was supported by key stakeholders including Toronto hospitals, the City of Toronto’s Social Development Department, the Toronto Office to Prevent Violence and various community organizations.^29,31^ Funding for both programs was obtained through municipal and federal government grants and private donors.^29,31^ Each program is staffed by social workers, trauma-informed clinicians, and peer support workers with lived experience. The program primarily focuses on providing trauma counseling, case management, social work, medical, and psychiatric follow-up. Furthermore, additional supports are provided to facilitate societal reintegration, including employment opportunities, housing, and education services.^29,31^ Services are initiated at the hospital where patients are connected with a case manager to develop personalized care plan. Following discharge, support continues to ensure access to services and assistance navigating the healthcare system. This support includes collaboration with partner organization and family engagement.^33^

## Cost Analysis of Hospital-based Violence Intervention Programs

Economic analysis often strengthens policy arguments for public health interventions. Acute traumatic care is costly both for health systems and patients. For healthcare systems, the estimated 1-year treatment costs of trauma care in the US are $75 210 per patient.^34^ While, in Canada, traumatic injury is associated with a 12.4% reduction in employment and a 19% decrease in earnings.^35^ Given the high rates of recidivism, these costs are likely underestimating the true economic burden of acute traumatic injury.^36^

Violence leads to significant reductions in life years, disability adjusted life years, or quality-adjusted life years (QALYs).^37^ Several studies have provided insights into the cost-effectiveness of HVIPs through incremental cost-effectiveness ratios (ICER), which measure the value of an intervention by comparing its additional cost to the additional health benefit.^38^ HVIPs at San Francisco General Hospital ICERs ranged from $5,685 to $7,724 per QALY gained,^39^ while Highland Hospital in San Francisco reported ICER of $2,941 per QALY.^40^ These values are well below the commonly accepted cost-effectiveness threshold of $100, 000 or $150, 000 per QALY in North America.^38^ These findings suggest that HVIPs provide valuable health benefits at justifiable costs. These studies did not consider societal costs, which would likely be favorable considering enhanced vocational resources and opportunities.

Emerging evidence has demonstrated that increased investment for marginalized patient populations may equate to better, more equitable outcomes after acute trauma. An example from the Canadian context is a study that examined a population-based cohort of patients with moderate to severe traumatic brain injuries managed in Ontario, Canada (where healthcare is universally accessible for Ontario residents). Investigators quantified the relationship between residence in racialized neighborhoods and healthcare expenditure/outcomes after brain injury and observed that patients residing in the most racialized communities incurred 18% more direct healthcare costs in the year following injury.^41^ However, despite the increased costs, patients from the most racialized communities had equivalent days spent alive and at home, equal access to rehabilitation, and comparable functional independence scores in the year after injury.^41^ This may suggest that targeted investments in healthcare may improve patient outcomes and potentially mitigate some of the disparities experienced by marginalized populations.

## Applying the Hospital-Based Violence Intervention Program Model Outside of The Trauma Context

The HVIP model raises an important question: Can this approach be applied beyond the trauma context and into the community? Community organizing frameworks, models, and processes such as the collective impact model, Alinsky-style organizing, socio-ecological framework, and community-based participatory research have been utilized within the academic literature and subsequently applied in various health initiatives, including promoting healthy lifestyles, sexual health, improving healthcare access, and managing chronic diseases.^42^ Therefore, this raises the question of how community organizing frameworks can be leveraged to contribute to safer communities in the context of violence.

A Canadian example is SafeTO’s 10-year strategic plan, which takes a holistic approach to addressing community safety challenges through both preventative (upstream) and responsive (downstream) programs.^43^ It integrates the socio-ecological framework to address health determinants at individual, community, and policy levels. It applies community-based participatory research to ensure interventions are co-developed with community members. Although the interventions themselves are individualized, SafeTO demonstrates a replicable process for engaging communities and integrating health frameworks. This suggests the potential for a standardized implementation process for HVIP models by drawing on what has worked in community health initiatives and expanding their application beyond the trauma setting.

## Challenges and Limitations

Several challenges limit the widespread implementation of HVIPs. One key barrier is the heterogeneity of healthcare systems across North America. In Canada, healthcare is administered at the provincial level, with each province operating its single-payer system.^44^ This can introduce variability in funding and program scalability across regions. Meanwhile, the United States operates under a multi-payer system, creating additional complexities that intertwine with SDoH.^44^ For example, insurance is often associated with employment or socio-economic status, and individuals at highest risk of violent injury often have limited or no insurance coverage.^45^ These structural differences may limit access to HIVP services and present challenges in adapting universal implementation models. Furthermore, it has been challenging to determine the population-based efficacy of a reinjury prevention program. To date, no robust population-based program for addressing upstream risk factors of violence has been implemented across large geographical regions in North America. Addressing these systemic and logistical challenges will be imperative to the applicability of HVIPs across diverse healthcare settings.

## Future Directions

Engaging patients while they are within the healthcare system presents a key opportunity, not only to treat their immediate medical needs but also to address the upstream social and community factors contributing to their condition. For many patients, this may be their only contact with the healthcare system. HVIPs capitalize on these “teachable moments.”^11,29^ Despite this, significant amounts of resources are dedicated to the prehospital and hospital phases,^7^ with less attention given to the discharge and immediate post-discharge periods. HVIPs fill this gap by engaging patients during recovery and providing longitudinal support after discharge.

In Canada, we have seen the successful implementation of HVIPs such as CEDVIP in Winnipeg as well as BRAVE and THRIVE in Toronto.^25,29,31^ HVIPs may also serve as models for broader healthcare delivery strategies, particularly in outpatient care and the management of chronic conditions for specialty populations impacted most by health disparities related to structural barriers. Through an equity lens, we understand the importance of this work for allowing equal access and equal opportunity for those to maximize their health, societal participation, and safety. However, making the policy case for allocating resources towards programming will require interdisciplinary collaborations across researchers, community advocates, clinicians, policymakers, patient partners, and many more in the public service space. From a research perspective, we will require constant evaluation of programming to assess effectiveness in prevention of reintervention, quality of life, and cost-utility analyses. HVIPs hold transformative potential, not only in reducing violence but also in addressing deeper upstream root causes and reshaping how healthcare systems engage with patients during these “teachable moments” that may be applied in other contexts outside of the trauma bay.
